# Cost-Effectiveness of Anticoagulation Treatment for Subclinical Device-Detected Atrial Fibrillation

**DOI:** 10.1001/jamanetworkopen.2026.17213

**Published:** 2026-06-08

**Authors:** Aleksi K. Winstén, Ville Langén, K.E. Juhani Airaksinen, Konsta Teppo

**Affiliations:** 1 Department of Mathematics and Statistics, University of Turku, Turku, Finland; 2 Faculty of Medicine, University of Turku, Turku, Finland; 3 Division of Medicine, Turku University Hospital and University of Turku, Turku, Finland; 4 Turku University Hospital and University of Turku, Turku, Finland; 5 Cardiac Unit, Department of Internal Medicine, Satasairaala, Pori, Finland; 6 Department of Internal Medicine, University of Turku, Turku, Finland

## Abstract

**Importance:**

Anticoagulation for stroke prevention in subclinical, device-detected atrial fibrillation (AF) remains an area of clinical equipoise, and its cost-effectiveness is unknown.

**Objective:**

To evaluate the cost-effectiveness of direct oral anticoagulant (DOAC) therapy in patients with device-detected AF.

**Design, Setting, and Participants:**

This economic evaluation was a cost-effectiveness analysis using a Markov model comparing initiation of DOAC therapy vs no anticoagulation over a 10-year time horizon. Base-case analyses modeled 10 000 patients per strategy with device-detected subclinical AF, with baseline characteristics and risks of stroke, bleeding, and mortality reflecting those observed in randomized clinical trials. The evaluation was conducted from the health system perspective, with treatment and event costs derived from Nordic health care data. The modeling was conducted on March 10, 2026.

**Exposure:**

The associations of DOAC therapy with the risk and severity of clinical events were incorporated into the analysis, based on a meta-analysis of trials evaluating DOAC therapy in subclinical AF. Probabilistic sensitivity analysis also considered the 95% CIs in the reported treatment effect sizes.

**Main Outcomes and Measures:**

Incremental quality-adjusted life-years (QALYs), costs, and the incremental cost-effectiveness ratio (ICER; cost difference per QALY gained) from a health system perspective, with 3% annual discounting of both costs and QALYs. Cost-effectiveness was assessed using a €50 000 per QALY willingness-to-pay threshold.

**Results:**

The mean age of the 20 000-person simulated cohort was 77 years. In the base case analysis, DOAC therapy was associated with an additional 0.016 QALYs and an incremental cost of €1676 per patient, resulting in an ICER of €105 293 per QALY. In probabilistic sensitivity analysis, DOAC therapy was cost-effective in 35% of simulations and dominated in 38%, with a mean QALY gain of 0.016 per patient, a mean incremental cost of €2883 per patient, and a mean ICER of €176 772. Probabilistic sensitivity analyses by CHA_2_DS_2_-VASc (congestive heart failure; hypertension; age ≥75 years; diabetes; prior stroke, transient ischemic attack, or thromboembolism; vascular disease; age 65-74 years; and sex category) score showed probabilities of cost-effectiveness of 31%, 41%, and 52% for patients with scores less than 4, of 4, and greater than 4, respectively.

**Conclusions and Relevance:**

This economic evaluation found that routinely initiating DOAC therapy in all patients with device-detected subclinical AF is unlikely to be cost-effective. Whether treatment is cost-effective in patients with very high CHA_2_DS_2_-VASc scores is uncertain.

## Key Points

**Question:**

Is anticoagulation for stroke prevention in patients with device-detected atrial fibrillation cost-effective?

**Findings:**

In this economic evaluation with a simulated cohort of 20 000 individuals, treatment with direct oral anticoagulants resulted in an additional 0.016 quality-adjusted life-years at an incremental cost of €1676 per patient over a 10-year simulation, corresponding to an incremental cost-effectiveness ratio of €105 293 per quality-adjusted life-year. The probability of cost-effectiveness was 35%, while there was a 38% probability of both lower quality-adjusted life-years and higher costs.

**Meaning:**

The findings of this study suggest that routine anticoagulation in all patients with device-detected atrial fibrillation may not be cost-effective and may represent low-value care.

## Teaser

This economic evaluation analyzes the cost-effectiveness of initiating direct oral anticoagulant therapy for patients with device-detected atrial fibrillation to reduce the risk of stroke.

## Introduction

The role of anticoagulation for stroke prevention in subclinical device-detected atrial fibrillation (AF) remains a subject of equipoise. Although the benefits of anticoagulation in patients with clinically diagnosed, electrocardiographically confirmed AF are well established, it remains unclear whether a comparable benefit is derived in situations in which cardiac devices incidentally detect short, asymptomatic AF episodes.^1^ The 2 pivotal randomized trials in this area showed that anticoagulation modestly reduced the risk of stroke at the expense of an increased risk of major bleeding.^2^ Moreover, our 2025 modeling study^3^ indicated that the net benefit of anticoagulant therapy, in terms of gained quality-adjusted life-years (QALYs), is minimal and uncertain. Nevertheless, if anticoagulation in this setting were demonstrated to be cost-effective, its use might be more justified despite the marginal clinical benefits. Thus, the current study aimed to evaluate whether direct oral anticoagulant (DOAC) therapy in patients with device-detected AF is cost-effective from a health system perspective.

## Methods

### Analytic Approach

A previously published Markov modeling study assessed the net benefit of DOAC therapy in subclinical device-detected AF in terms of QALYs.^3^ In the current study, this previously published model was updated to address the question of cost-effectiveness. Cost-effectiveness was measured as incremental QALYs, costs, and the incremental cost-effectiveness ratio (ICER; cost per QALY gained) for initiating DOAC therapy compared with the decision to withhold DOACs. The Markov model consists of multiple health states individuals can move between based on specific transition probabilities (Table 1).^2,4,5,6,7,8,9^ The model used a 1-month cycle length, and all event probabilities were transformed into 1-month probabilities (Table 1). The simulation was run for a 10-year period with 10 000 individuals in both decision groups. eFigure 1 in Supplement 1 depicts the Markov model and the health states. The event rates and probabilities used, along with their sources, are presented in Table 1. The study follows the Consolidated Health Economic Evaluation Reporting Standards (CHEERS) reporting guideline. According to Finnish legislation on medical research, ethical approval was deemed unnecessary, as this study utilized an analytical model based on publicly available data without collecting new data or accessing patient information. In the interest of research reproducibility, we have deposited the codes of the Markov model in the Zenodo repository.^10^

**Table 1.** Model Input Parameters: Event Rates, Quality of Life, and Event and Mortality Risk^a^

Event	Input	Source
**Event rates, untreated rate/100 person-years**		
Ischemic stroke	1.05^b^	Kirchhof et al,^4^ 2023; Healey et al,^5^ 2023; McIntyre et al,^2^ 2023
Major bleeding	1.06^b^	
Hemorrhagic stroke	0.18^c^	
Other intracranial bleeding	0.15^c^	
Extracranial major bleeding	0.73^d^	
Death	4.26^b^	
Clinical atrial fibrillation	7.50^b^	
**Outcomes associated with anticoagulation, relative risk (95% CI)**		
Ischemic stroke	0.68 (0.50-0.92)	McIntyre et al,^2^ 2023
Major bleeding	1.62 (1.05-2.50)	
**Base-case quality of life**		
Well without events from age 77-80 y	0.794	Burström et al,^6^ 2006
Well without events from age 80-87 y	0.733	
**Quality-adjusted life-year ratios**		
Mild disability until 6 mo after event	0.88	Luengo-Fernandez et al,^7^ 2013
Mild disability from 6 mo after event	0.89	
Moderate disability until 6 mo after event	0.60	
Moderate disability from 6 mo after event	0.73	
Severe disability until 6 mo after event	0.16	
Severe disability from 6 mo after event	0.45	
Death	0	
**Event risks, 1-y probability**		
Mortality		
After ischemic stroke	0.14	Sennfält et al,^8^ 2019
After any intracranial bleeding	0.16	
Event risks after developing clinical AF		
Ischemic stroke	0.025	Teppo et al,^9^ 2022
Hemorrhagic stroke	0.005	
Other intracranial bleeding	0.005	
Extracranial major bleeding	0.028	
Death	0.110	

Abbreviation: AF, atrial fibrillation.

^a^ Rates and probabilities were transformed to 1-month probabilities for the model.

^b^ Average rate from the NOAH-AFNET 6 and ARTESiA trials.

^c^ Derived from the ARTESiA trial.

^d^ Calculated by subtracting the intracranial bleeding rate from the total major bleeding rate.

As a base-case patient for the analysis, aligning with the mean age and characteristics of the NOAH-AFNET 6 and ARTESiA trial populations, we used a 77-year-old patient without a prior history of clinical AF and applied the average untreated stroke, bleeding, and mortality rates from the trials.^2^ Patients were described in the original trial as European or North American. Our model focused on ischemic strokes, major bleeding, and mortality, given that these are the main outcomes to consider when evaluating the benefits and harms of anticoagulant therapy. Other events, such as myocardial infarctions and pulmonary embolisms, were excluded from the model because their incidence did not change with anticoagulation in the trials and their impact on quality of life varies considerably.

### Outcomes of Anticoagulation

In the main analysis, pooled point estimates from the meta-analysis of the 2 trials^2^ were used to model the outcomes associated with anticoagulation for stroke and major bleeding. Anticoagulation was not associated with a significant effect size on mortality in either the individual trials or their combined analysis, and therefore, identical baseline mortality rates were applied to both the anticoagulation and no-anticoagulation groups.^2^ As in the previously published model and in line with evidence that nonintracranial bleeding events predominate among DOAC-related bleeding events, we assigned an 80% weight to nonintracranial bleeding events for the increase in bleeding associated with DOACs, while keeping the overall increase in major bleeding consistent with the meta-analysis results.^11^ The impact of different weights was examined in sensitivity analyses. Following all major bleeding events in patients receiving DOACs, treatment was temporarily paused for 1 month in the model. Otherwise, full adherence to the initiated therapy was assumed. The initial 30-day mortality related to stroke and bleeding events was included in the model. In addition, ischemic stroke and all intracranial bleeding events were assumed to increase mortality during the first year following the event, with these mortality probabilities derived from a large Swedish population-based study.^8^ Probabilities for the severity of stroke and bleeding events in the anticoagulation and nonanticoagulation groups of the model were approximated based on previous observations in patients with and without anticoagulation as well as data from the NOAH-AFNET 6 and ARTESiA trials (Table 2).^3,5,8,12,13,14,15,16,17,18,19,20,21,22,23,24,25,26^

**Table 2.** Model Input Parameters: Event Severity

Event	Probability		Source
	No DOAC	DOAC	
**Ischemic stroke**			
Death	0.187	0.120	Sennfält et al,^8^ 2019; Vinding et al,^12^ 2022; Healey et al,^5^,2023; Walraven et al,^13^ 2002; Fang et al,^14^ 2012; Diener et al,^15^ 2024
Severe disability	0.156	0.124	
Moderate disability	0.220	0.247	
Mild disability	0.437	0.509	
**Hemorrhagic stroke**			
Death	0.175	0.305	Rosand et al,^16^ 2004; Fang et al,^17^ 2007; Healey et al,^5^ 2023; Giugliano et al,^18^ 2013; Skaistis et al,^19^ 2015; Toyoda et al,^20^ 2022
Severe disability	0.172	0.206	
Moderate disability	0.301	0.190	
Mild disability	0.352	0.293	
**Other intracranial bleeding**			
Death	0.157	0.176	Fang et al,^14^ 2012; Weimer et al,^21^ 2017; Poon et al,^22^ 2021; Giugliano et al,^18^ 2013; Healey et al,^5^ 2023; Skaistis et al,^19^ 2015; Gaist et al,^23^ 2017
Severe disability	0.179	0.237	
Mild disability	0.332	0.293	
No disability	0.332	0.293	
**Extracranial major bleeding**			
Death	0.025	0.035	Fang et al,^17^ 2007; Walraven et al,^13^ 2002; Vora et al,^24^ 2020; Chornenki et al,^25^ 2023; Giugliano et al,^18^ 2013; Healey et al,^5^ 2023; Skaistis et al,^19^ 2015; Gómez-Outes et al,^26^ 2021
Severe disability	0.008	0.007	
Mild disability	0.058	0.056	
No disability	0.909	0.902	

Abbreviation: DOAC, direct oral anticoagulant

### Development of Clinical AF

Patients with subclinical AF often develop clinical AF, and their subsequent treatment is guided by existing clinical practice guidelines for AF management, usually including anticoagulation.^2^ As in the previously published model, all patients who developed overt AF were assumed to have the average stroke, bleeding, and mortality rates of contemporary real-life patients with clinical AF, regardless of whether they had received anticoagulation in the model prior to developing clinical AF.^9^ Thus, after the onset of clinical AF, prognosis was modeled similarly for both decision groups. In these patients, event severity probabilities were based on those observed in patients receiving anticoagulation.

### Utility Weights

Baseline QALY weights were derived from age-specific utility values of the general Swedish population reported by Burström et al.^6^ Health-related quality-of-life decrements associated with ischemic stroke and intracranial hemorrhage were incorporated using estimates from Luengo-Fernández et al,^7^ which report utility values stratified by event severity. In our model, a multiplicative approach was used to derive utility values, so that postevent QALY weights were calculated by multiplying an individual’s pre-event utility by the ratio of utility in patients with the relevant event severity to that of control patients, as reported in the study. For the rare major extracranial bleeding events resulting in permanent disability, the same approach was applied, with utility decrements assigned according to the severity of the resulting disability.

### Statistical Analysis

The study used a health system perspective, considering direct medical costs of care and treatment for both health care practitioners and patients, while excluding nonmedical costs and productivity losses, since most patients with device-detected AF are well above the typical working age.^27^ In the current study, health care costs related to events and DOAC therapy were added to the previously published model, derived from recently published Nordic health care data (eTable 1 in Supplement 1).^28,29,30^ Death was considered a one-time cost, whereas all other costs were modeled as monthly costs. Costs related to stroke-related death were based on recent Finnish health care data.^28^ Other stroke-related costs by event severity were derived from recently published Swedish data by Lekander et al.^30^ For other intracranial and extracranial bleeding events, data stratified by severity were unavailable, and therefore, a uniform cost estimate based on Danish data was applied.^29^ In years after the first year, only moderate and severe disability following stroke or bleeding were assumed to generate ongoing costs. For these subsequent years, the same annual cost was applied to all cases with severe disability related to bleeding, as no data were available to distinguish between intracranial and extracranial bleeding costs. Given the similarity of health care systems across Nordic countries, cross-country cost estimates were considered acceptable. The cost of DOAC therapy, including associated laboratory tests and health care visits, was set at €50 per month based on average prices from the Finnish Medicinal Products Database (to convert euros to US dollars, multiply by 1.17),^31^ slightly lower than estimates used in recent cost-effectiveness analyses of patients with AF across European countries.^32^ In sensitivity analyses, costs of DOAC therapy were varied according to the lowest and highest reported DOAC costs. All costs were adjusted to 2025 price levels.

A 3% discount rate was applied to both costs and QALYs, reflecting the standard discount rates used in previously published economic evaluations in the Nordic context.^33^ Different discount rates were explored in sensitivity analyses. In the main analysis, a commonly applied willingness-to-pay (WTP) threshold of €50 000 per QALY was used.^34^ Moreover, the impact of varying WTP thresholds on the probability of cost-effectiveness was assessed in probabilistic sensitivity analyses and visualized using a cost-effectiveness acceptability curve.

Sensitivity analyses were performed to address parameter uncertainty in the model. In probabilistic sensitivity analyses, the 95% CIs of the reported estimates for stroke, major bleeding, and mortality were used (relative risks of 0.68 [95% CI, 0.50-0.92], 1.62 [95% CI, 1.05-2.50], and 1.08 [95% CI, 0.96-1.21], respectively),^2^ assuming a log-normal distribution. Unfortunately, the trials did not report measures of uncertainty for the event rates, and therefore, no arbitrary variation was applied to the untreated event rates in the probabilistic sensitivity analyses. The model was run for a 10-year period with 2000 iterations of sampled treatment effect estimates, with 1000 patients in both decision groups. The number of individuals in each decision group in the probabilistic sensitivity analysis was smaller than in the main analysis to ensure computational feasibility and was considered sufficient to provide stable results per iteration. The mean QALY difference and incremental costs per patient were calculated, along with the mean ICER and the proportion of iterations resulting in cost-effective outcomes according to the €50 000 WTP threshold. Additionally, the results were visualized across different WTP thresholds using a cost-effectiveness acceptability curve.

Additionally, a substudy of the ARTESiA trial suggested that those with higher risk scores had a numerically lower risk of stroke when treated with DOACs compared with aspirin.^35^ However, this finding was not statistically significant, not adjusted for multiplicity, and a similar pattern was not observed in the NOAH-AFNET 6 trial.^36^ Nevertheless, we conducted exploratory deterministic sensitivity analyses stratified by CHA_2_DS_2_-VASc (congestive heart failure; hypertension; age ≥75 years; diabetes; prior stroke, transient ischemic attack, or thromboembolism; vascular disease; age 65-74 years; and sex category) categories (<4, 4, >4), using event rates and point estimates of treatment effects for stroke and bleeding as reported in the ARTESiA substudy.^3^ We also performed probabilistic sensitivity analyses by these risk categories, considering the 95% CIs of the reported treatment effect sizes. Since effects on mortality were not reported by risk categories, the same estimates for mortality were used across all categories (eTable 2 in Supplement 1). Of note, because none of the multiple interactions tested between treatment effects and risk categories in the ARTESiA and NOAH-AFNET 6 substudies reached statistical significance, these analyses are regarded as hypothesis generating only. Moreover, because treatment effects are based solely on the ARTESiA trial, the categorized analysis does not reflect sections of our main analysis, which used treatment effects from the meta-analysis of the 2 trials. In probabilistic sensitivity analyses by risk categories, the model was run over a 10-year period with 2000 iterations of sampled treatment effect estimates, with 1000 patients in each decision arm.

Moreover, 1-way sensitivity analyses were conducted for selected parameters that may exhibit meaningful variation to assess the robustness of the results and identify key drivers of the model outcomes. First, untreated stroke, bleeding, and mortality rates were varied by ±50%. Second, the proportion of increased nonintracranial bleeding events associated with DOAC therapy was varied from the base-case assumption of 80% to 70% and 90%. Third, the associations of DOACs with stroke and bleeding were varied using the point estimates reported in the ARTESiA and NOAH-AFNET 6 trials, in contrast to the meta-analytic estimates applied in the main analysis. Fourth, the discount rate was varied to 0% and 5%.

The modeling was conducted on March 10, 2026. All analyses were performed R version 4.0.5 (R Project for Statistical Computing).

## Results

The model was run with 10 000 patients with anticoagulation and 10 000 patients without anticoagulation. The patients had a mean age of 77 years and characteristics similar to those of patients in randomized trials of anticoagulation in subclinical AF.^2^ In the base-case analysis, initiating DOACs resulted in 227 fewer ischemic strokes (21.8% decrease), 71 fewer deaths (1.4% decrease), and 339 more major bleeding events (42.1% increase) compared with no anticoagulation over a 10-year simulation period. These differences resulted in an additional 0.016 QALYs at an incremental cost of €1676 per patient, yielding an ICER of €105 293.

**Figure 1.**
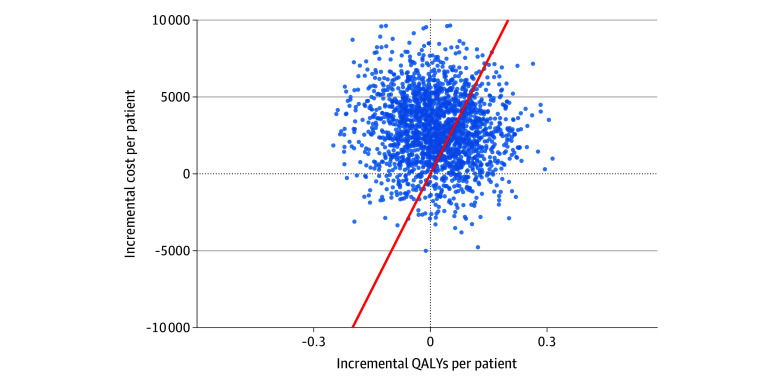
Incremental Cost-Effectiveness Plane of Incremental Costs and Quality-Adjusted Life-Years (QALYs) per Patient With Direct Oral Anticoagulation Therapy Compared With Withholding Anticoagulation The red line indicates the willingness-to-pay threshold of €50 000. To convert euros to US dollars, multiply by 1.17.

**Figure 2.**
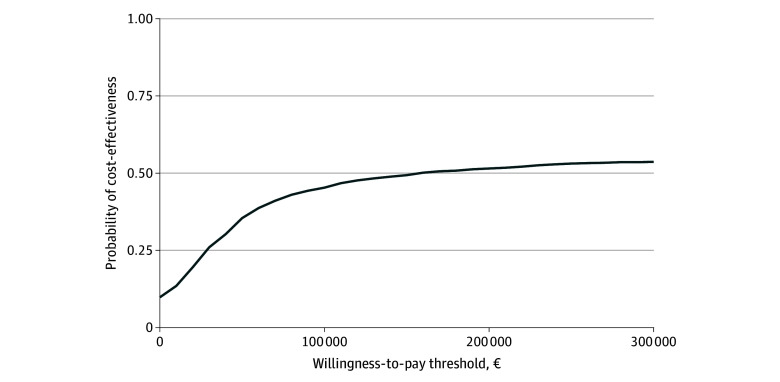
Cost-Effectiveness Acceptability Curve for Direct Oral Anticoagulation Therapy in the Probabilistic Sensitivity Analysis To convert euros to US dollars, multiply by 1.17.

In probabilistic sensitivity analyses, using a WTP threshold of €50 000 per QALY, DOAC therapy was cost-effective in 35% of simulations, with a mean QALY gain of 0.016, a mean cost difference of €2883, and a mean ICER of €176 772 (Figure 1). Moreover, the probability that DOAC therapy resulted in both less QALYs and more costs was 38%. The probability of cost-effectiveness remained below 50% at WTP thresholds up to €150 000 and was only slightly greater than 50% between €150 000 and €300 000 (Figure 2).

In the exploratory analyses stratified by CHA_2_DS_2_-VASc score, patients with a score of less than 4 had a mean QALY gain of 0.014 per patient and a mean cost increase of €1778.16 per patient, resulting in an ICER of €127 238.12. Among patients with a CHA_2_DS_2_-VASc score of 4, the mean QALY gain was 0.032 per patient, and the mean cost increase was €823.29 per patient, corresponding to an ICER of €25 523.07. In patients with a CHA_2_DS_2_-VASc score greater than 4, DOAC therapy resulted in a mean QALY gain of 0.039 per patient and a mean cost increase of €469.54 per patient, yielding an ICER of €11 895.08.

Consistent with these results, when uncertainty in the estimated treatment effects was considered in the probabilistic sensitivity analysis, DOAC therapy among patients with a CHA_2_DS_2_-VASc score less than 4 was associated with a mean QALY gain of 0.009 per patient and a mean cost increase of €3214 per patient, resulting in an ICER of €340 916 and a 31% probability of cost-effectiveness. Among patients with a CHA_2_DS_2_-VASc score of 4, DOAC therapy resulted in a mean QALY gain of 0.023 per patient and a mean cost increase of €2344 per patient, with an ICER of €102 983 and a 41% probability of cost-effectiveness. In patients with a CHA_2_DS_2_-VASc score greater than 4, DOAC therapy resulted in a mean QALY gain of 0.035 per patient and a mean cost increase of €1569 per patient, with an ICER of €44 595 and a 52% probability of cost-effectiveness. Cost-effectiveness acceptability curves showed that the probability of cost-effectiveness remained less than 50% in patients with CHA_2_DS_2_-VASc scores less than 4 across WTP thresholds of €0 to €300 000, in those with a score of 4 at WTP thresholds less than €100,000, and in those with scores greater than 4 at WTP thresholds less than approximately €50 000 (eFigures 2-4 in Supplement 1).

In other sensitivity analyses, the ICER was less than the WTP threshold of €50 000 per QALY when baseline stroke risk was individually increased by 50%, major bleeding risk was decreased by 50%, monthly cost of DOAC therapy was €30, and when the treatment effects from the ARTESiA trial were applied. In all other sensitivity analyses, the ICER exceeded the WTP threshold of €50 000 per QALY (Table 3).

**Table 3.** Results of the Sensitivity Analyses^a^

Input	Gained QALYs	Cost difference per patient, €	ICER, €
Baseline stroke rate^b^			
1.575 (50% Higher)	0.036	565.19	15 514.91^c^
0.525 (50% Lower)	−0.002	3649.43	Dominated
Baseline bleed rate^b^			
1.59 (50% Higher)	−0.001	2159.04	Dominated
0.53 (50% Lower)	0.032	1157.30	36 053.01^c^
Baseline death rate^b^			
6.39 (50% Higher)	0.009	1219.16	133 865.61
2.13 (50% Lower)	0.019	1787.51	95 862.40
Proportion of nonintracranial bleeding			
70%	0.005	1844.38	341 494.22
90%	0.022	1337.43	61 286.44
Discount rate			
0%	0.023	1381.42	61 022.34
5%	0.021	1310.54	61 386.87
DOAC therapy monthly cost			
€30 (40% Lower)	0.016	104.77	6481.73^c^
€70 (40% Higher)	0.016	3247.34	204 004.02
Treatment effects on stroke, RR^d^			
0.79 (16% Higher)	0.013	1563.85	119 951.60
0.62 (9% Lower)	0.026	1012.53	38 548.43
Treatment effects on bleeding, RR^d^			
2.10 (30% Higher)	0.010	2057.22	210 095.32
1.36 (16% Lower)	0.035	984.84	28 390.41^c^
Treatment effects from trials, RR^e^			
NOAH-AFNET 6: stroke, 0.79; major bleeding, 2.10	−0.015	3227.11	Dominated
ARTESiA: stroke, 0.62; major bleeding, 1.36	0.033	1013.89	31 080.49^b^

Abbreviations: DOAC, direct oral anticoagulant; ICER, incremental cost-effectiveness ratio; QALY, quality-adjusted life year; RR, relative risk.

^a^ Positive values indicate QALY gains and increased costs with DOAC therapy, whereas negative values indicate QALY losses and cost reductions with treatment. A dominated strategy refers to higher costs and fewer QALYs with DOAC therapy. To convert euros to US dollars, multiply by 1.17.

^b^ Per 100 patient-years; baseline stroke, bleeding, and mortality rates in the main model were 1.05, 1.06, and 4.36, respectively.

^c^ ICER below the willingness-to-pay threshold of €50 000 per QALY.

^d^ Treatment effects were varied individually based on the point estimates from the NOAH-AFNET 6 and ARTESiA trials.

^e^ Both stroke and bleeding treatment effects were varied concurrently according to the point estimates from the NOAH-AFNET 6 and ARTESiA trials.

## Discussion

In this cost-effectiveness analysis, the minimal QALY gains and an ICER of approximately €100 000 in the main analysis suggest that routine DOAC therapy for all patients with device-detected AF is not cost-effective. Notably, while the probabilistic sensitivity analysis showed only a 35% probability of cost-effectiveness, there was also a 38% probability that DOAC therapy would be associated with both higher costs and lower QALYs.

Current clinical practice guidelines on AF management give a cautious level IIb recommendation that DOACs may be considered in selected patients with subclinical AF who are not at increased bleeding risk, acknowledging the uncertainty in the balance of benefits and harms.^37^ In these patients, the average absolute stroke risk is relatively low (around 1% annually), and although DOAC therapy provides some risk reduction, this benefit is modest and offset by an increased risk of bleeding, as observed particularly in the placebo-controlled NOAH-AFNET 6 trial.^4^ Consistent with this, our previous modeling work suggested that the net clinical benefit is uncertain and, if present, likely to be small and not clinically meaningful. The present study complements these findings by demonstrating that routine DOAC therapy in patients with device-detected AF is unlikely to be cost-effective. The estimated QALY gains are minimal and the ICERs exceed commonly accepted thresholds.

While treatment was very unlikely to be cost-effective in those with low CHA_2_DS_2_-VASc scores, our results suggest an approximately 50% probability of cost-effectiveness in those with very high scores (>4) at WTP threshold of €50 000, with the probability increasing slightly at higher WTP thresholds. However, given the considerable uncertainty in the evidence on benefits and harms across risk categories, particularly that improved stroke prevention with DOACs in higher-risk patients was not statistically significant and observed only in the ARTESiA trial but not in the NOAH-AFNET 6, these stratified analyses should be considered hypothesis generating only.

In the 1-way sensitivity analyses, treatment appeared cost-effective when the baseline stroke rate was increased in isolation by 50% or when the bleeding risk was decreased in isolation by 50%. However, such variations are of limited clinical plausibility, as patients at higher risk of stroke are typically also at increased risk of bleeding due to shared risk factors.^38^ Moreover, if the cost of DOAC therapy continues to decrease with the introduction of generic formulations, treatment may be cost-effective at very low monthly costs of €30. Treatment appeared cost-effective when the treatment effects from the ARTESiA trial were applied. However, this finding should be interpreted with caution, as ARTESiA used aspirin as the comparator, whereas the NOAH-AFNET 6 trial used placebo. Indeed, when treatment effects from NOAH-AFNET 6 were applied, the treatment strategy was dominated, being associated with both lower QALYs and higher costs.

It is notable that, across the results including all sensitivity analyses, the QALY difference associated with treatment ranged from approximately 1 quality-adjusted week lost to 2 weeks gained, indicating minimal and likely clinically negligible benefits for most patients.^39^ Moreover, the very high ICER values observed in this study were primarily driven by these small average gains in QALYs. Because QALY gains form the denominator of the ICER, very small absolute differences in effectiveness can lead to very large ICER estimates. Together, these findings underscore that meaningful clinical effectiveness is a key prerequisite for an intervention to achieve favorable cost-effectiveness.

Of note, our model starts from the setting when subclinical AF is already detected and does not account for the resources required to detect and manage atrial high-rate episode alerts, including screening, confirmation of arrhythmia, and adjustment of device settings. In practice, this process preceding the decision to initiate anticoagulation inevitably increases overall costs related to subclinical AF, further reducing cost-effectiveness. Taken together, the combination of marginal and uncertain health gains, increased costs, and the potential for harm suggests that systematic screening and treatment of subclinical AF in patients with cardiac devices is likely to represent low-value care by definition.^40^ Indeed, a default strategy of deactivating atrial high-rate episode alerts in cardiac implantable devices and activating them only when clinically indicated could potentially reduce health care resource use without adversely affecting net clinical outcomes.

### Limitations

Our study is subject to the inherent limitations of cost-effectiveness analyses and mathematical modeling of complex real-world scenarios, including parameter uncertainty and the omission of other potentially meaningful events. The model focused on the most important outcomes affected by the decision to initiate anticoagulation. That said, the model excluded nonmajor bleeding and assumed full treatment adherence, both of which may lead to an overestimation of the benefits of anticoagulation compared to real-world settings. Moreover, our analysis utilized cost estimates derived from Nordic data and although costs are broadly similar across many European countries, they can vary substantially between health care systems.^32,41^ While our model was based on data from patients enrolled in randomized clinical trials and suggests that routine DOAC therapy is not cost-effective, further studies are needed to better identify real-world patient subgroups who may derive net benefit. Due to limited data, the probabilistic sensitivity analyses considered only uncertainty in the treatment effects of DOACs. Moreover, while the model produced plausible results in one-way sensitivity analyses, such as improved cost-effectiveness with lower DOAC costs or higher baseline stroke risk, supporting its internal validity, external validation was not feasible due to the lack of published quality-of-life and cost data specifically in patients with subclinical AF. Nevertheless, external validity is supported by the agreement between the previously reported cumulative incidence of events from the same model and the incidence curves reported in the trials.^3,42^

## Conclusions

In conclusion, this economic evaluation suggests that routine DOAC therapy for all patients with device-detected AF is unlikely to be cost-effective. Treatment of incidentally detected subclinical AF in patients with cardiac devices may represent low-value care, and further research is needed to identify those who are most likely to derive meaningful clinical benefit and for whom treatment may be cost-effective.

## Notes

Supplement 1. **eTable 1. **Event-Related Health Care Costs **eTable 2.** Model Input Parameters Used in the Sensitivity Analyses for Patients With CHA2DS2-VASc Scores of Less Than 4, 4, and More Than 4 **eFigure 1.** Structure in the Markov Decision Model Cycle **eFigure 2.** Cost-Effectiveness Acceptability Curve for DOAC Therapy in the Probabilistic Sensitivity Analysis in Patients With CHA_2_DS_2_-VASc Score Less Than 4 **eFigure 3.** Cost-Effectiveness Acceptability Curve for DOAC Therapy in the Probabilistic Sensitivity Analysis in Patients With CHA_2_DS_2_-VASc Score of 4 **eFigure 4.** Cost-Effectiveness Acceptability Curve for DOAC Therapy in the Probabilistic Sensitivity Analysis in Patients With CHA_2_DS_2_-VASc Score Greater Than 4

Supplement 2.
Data Sharing Statement
