# Supplementary material for: Cost-Effectiveness of Anticoagulation Treatment for Subclinical Device-Detected Atrial Fibrillation
Source: JAMA Netw Open. 2026 Jun 8;9(6):e2617213. doi: 10.1001/jamanetworkopen.2026.17213 (PMC13247805; doi:10.1001/jamanetworkopen.2026.17213)
Supplement: Supplement 1. — eTable 1. Event-Related Health Care Costs eTable 2. Model Input Parameters Used in the Sensitivity Analyses for Patients With CHA2DS2-VASc Scores of Less Than 4, 4, and More Than 4 eFigure 1. Structure in the Markov Decision Model Cycle eFigure 2. Cost-Effectiveness Acceptability Curve for DOAC Therapy in the Probabilistic Sensitivity Analysis in Patients With CHA2DS2-VASc Score Less Than 4 eFigure 3. Cost-Effectiveness Acceptability Curve for DOAC Therapy in the Probabilistic Sensitivity Analysis in Patients With CHA2DS2-VASc Score of 4 eFigure 4. Cost-Effectiveness Acceptability Curve for DOAC Therapy in the Probabilistic Sensitivity Analysis in Patients With CHA2DS2-VASc Score Greater Than 4 [file jamanetwopen-e2617213-s001.pdf]

Supplemental Online Content

Winstén AK, Langén V, Airaksinen KJ, Teppo K. Cost-effectiveness of anticoagulation treatment for subclinical device-detected atrial fibrillation. *JAMA Netw Open.* 2026;9(6):e2617213. doi:10.1001/jamanetworkopen.2026.17213

eTable 1. Event-Related Health Care Costs

eTable 2. Model Input Parameters Used in the Sensitivity Analyses for Patients With CHA2DS2-VASc Scores of Less Than 4, 4, and More Than 4

eFigure 1. Structure in the Markov Decision Model Cycle

eFigure 2. Cost-Effectiveness Acceptability Curve for DOAC Therapy in the Probabilistic Sensitivity Analysis in Patients With CHA_2DS_2-VASc Score Less Than 4

eFigure 3. Cost-Effectiveness Acceptability Curve for DOAC Therapy in the Probabilistic Sensitivity Analysis in Patients With CHA_2DS_2-VASc Score of 4

eFigure 4. Cost-Effectiveness Acceptability Curve for DOAC Therapy in the Probabilistic Sensitivity Analysis in Patients With CHA_2DS_2-VASc Score Greater Than 4

This supplemental material has been provided by the authors to give readers additional information about their work.

© 2026 Winstén AK et al. *JAMA Network Open*.

eTable 1. Event-Related Health Care Costs

Disability level	Ischemic stroke	Hemorrhagic stroke	Other intracranial bleeding	Extracranial bleeding
**1st year costs**				
No disability	NA	NA	22020	17318
Mild	6259	16880	22020	17318
Moderate	52721	52721	NA	NA
Severe	100516	65906	22020	17318
Death	14192	16508	22020	17318
**Subsequent years costs**				
No disability	NA	NA	0	0
Mild	0	0	0	0
Moderate	55425	79006	NA	NA
Severe	102506	75798	75798	75798

All costs are in euros and adjusted to 2025 price levels.

© 2026 Winstén AK et al. *JAMA Network Open*.

eTable 2. Model Input Parameters Used in the Sensitivity Analyses for Patients With CHA$_2$DS$_2$-VASc Scores of Less Than 4, 4, and More Than 4

**CHA$_2$DS$_2$-VASc <4**	**Untreated rate per 100 patient-years**
Ischemic stroke	0.99 [a]
Major bleeding	1.11 [a]
Death	3.40 [b]
**Effect of anticoagulation**	**Relative risk (95% CI)**
Ischemic stroke	0.87 (0.50-1.52) [c]
Major bleeding	1.27 (0.80-2.03) [c]
Mortality	1.08 (0.96–1.21) [d]
**CHA$_2$DS$_2$-VASc =4**	**Untreated rate per 100 patient-years**
Ischemic stroke	0.93 [a]
Major bleeding	1.00 [a]
Death	3.40 [b]
**Effect of anticoagulation**	**Relative risk (95% CI)**
Ischemic stroke	0.63 (0.32-1.27) [c]
Major bleeding	1.31 (0.75-2.29) [c]
Mortality	1.08 (0.96–1.21) [d]
**CHA$_2$DS$_2$-VASc >4**	**Untreated rate per 100 patient-years**
Ischemic stroke	1.78 [a]
Major bleeding	1.18 [a]
Death	5.60 [b]
**Effect of anticoagulation**	**Relative risk (95% CI)**
Ischemic stroke	0.44 (0.25-0.77) [c]
Major bleeding	1.48 (0.89-2.45) [c]
Mortality	1.08 (0.96–1.21) [d]

All other parameters and the proportions of bleeding subtypes were consistent with those in the main analysis.

[a] Average nonanticoagulated rate of the NOAH and ARTESiA trials

[b] All-cause mortality in the NOAH trial (ARTESiA did not report mortality rates in different risk score categories)

[c] Derived from the ARTESiA trial data

[d] Derived from the meta-analysis of NOAH and ARTESiA trials

© 2026 Winstén AK et al. *JAMA Network Open*.

eFigure 1. Structure in the Markov Decision Model Cycle

The model was run separately for patients with and without anticoagulation with different transition probabilities.

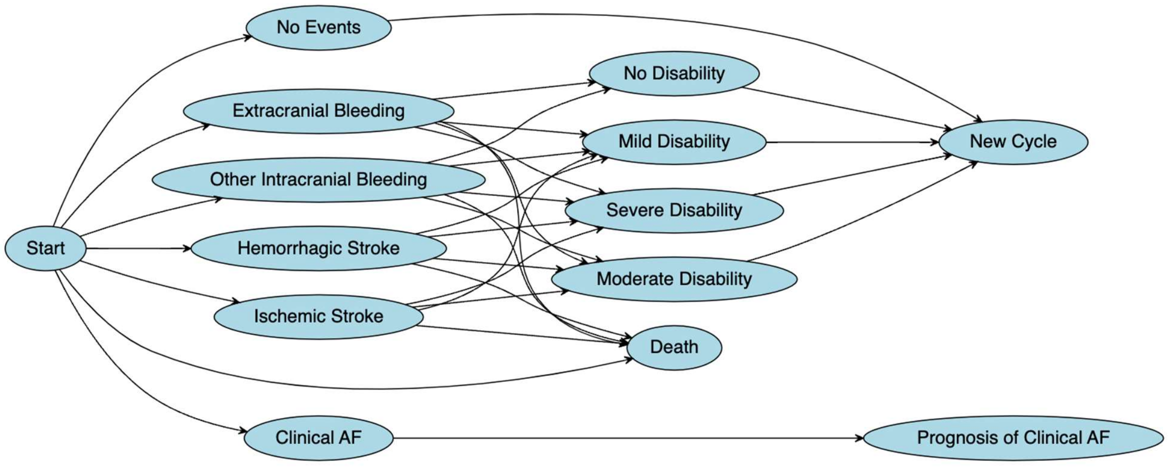

© 2026 Winstén AK et al. *JAMA Network Open*.

eFigure 2. Cost-Effectiveness Acceptability Curve for DOAC Therapy in the Probabilistic Sensitivity Analysis in Patients With CHA_2DS_2-VASc Score Less Than 4

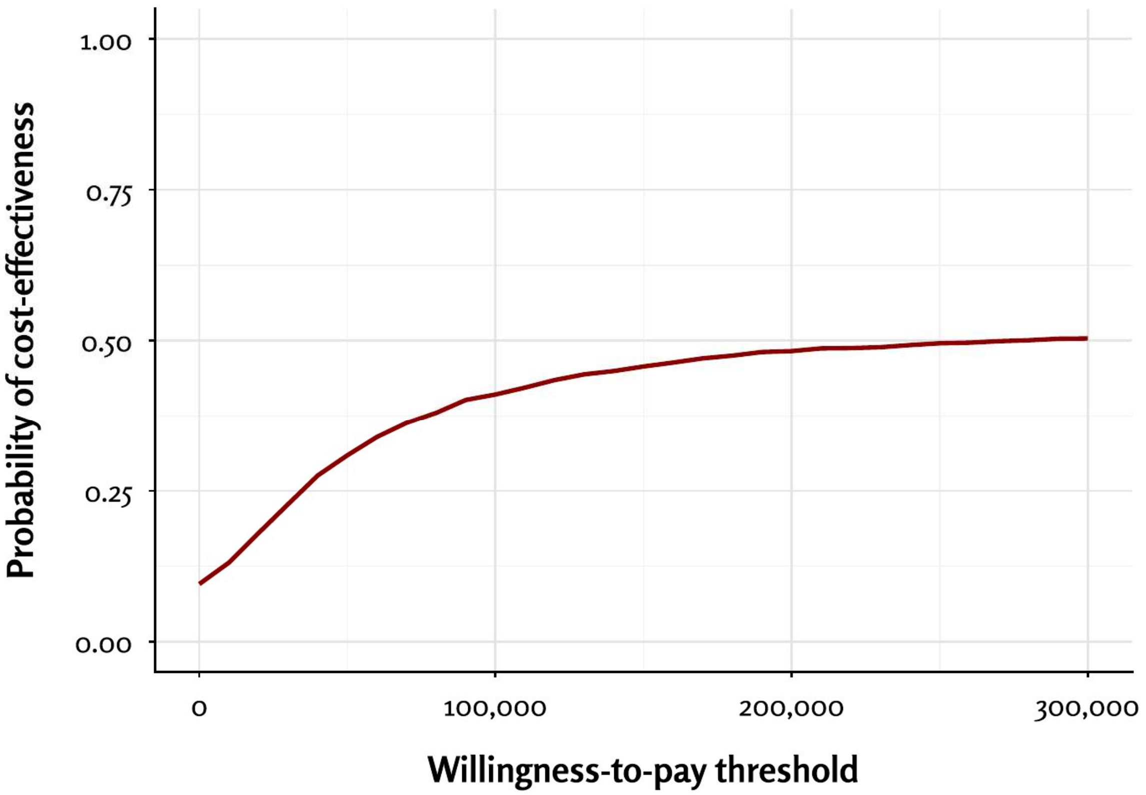

© 2026 Winstén AK et al. *JAMA Network Open*.

eFigure 3. Cost-Effectiveness Acceptability Curve for DOAC Therapy in the Probabilistic Sensitivity Analysis in Patients With CHA_2DS_2-VASc Score of 4

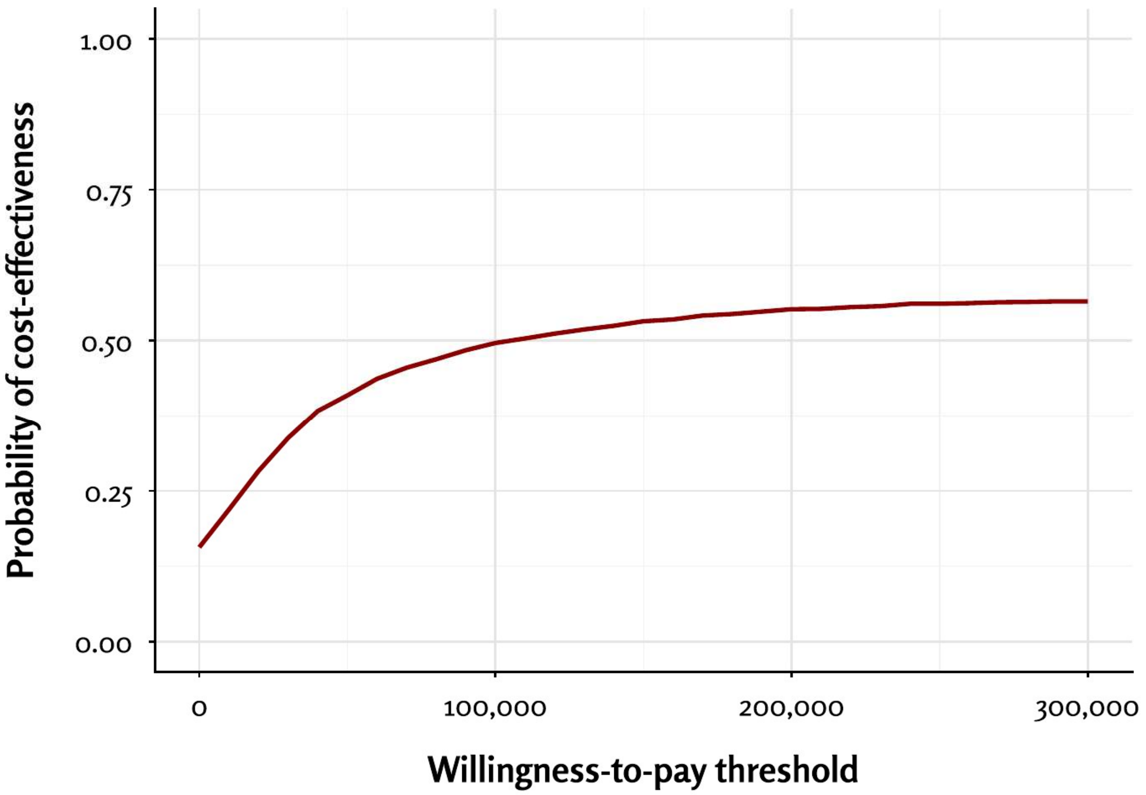

© 2026 Winstén AK et al. *JAMA Network Open*.

eFigure 4. Cost-Effectiveness Acceptability Curve for DOAC Therapy in the Probabilistic Sensitivity Analysis in Patients With CHA_2DS_2-VASc Score Greater Than 4

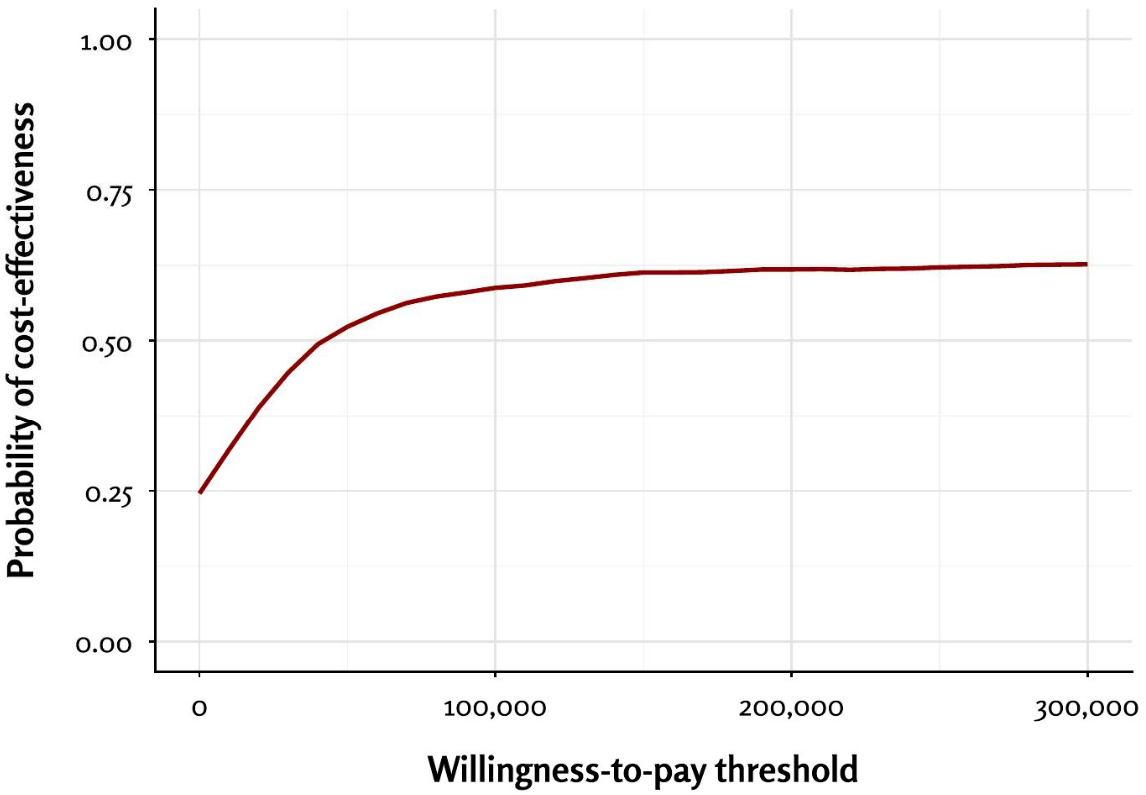

© 2026 Winstén AK et al. *JAMA Network Open*.
