# Supplementary material for: Cost-Effectiveness of Anticoagulation Treatment for Subclinical Device-Detected Atrial Fibrillation
Source: JAMA Netw Open. 2026 Jun 8;9(6):e2617213. doi: 10.1001/jamanetworkopen.2026.17213 (PMC13247805; doi:10.1001/jamanetworkopen.2026.17213)
Supplement: Supplement 2. — Data Sharing Statement [file jamanetwopen-e2617213-s002.pdf]

Data Sharing Statement

Winstén. Cost-Effectiveness of Anticoagulation Treatment for Subclinical Device-Detected Atrial Fibrillation. *JAMA Netw Open*. Published June 08, 2026.
doi:10.1001/jamanetworkopen.2026.17213

Data
Data available: Yes
Data types: Data (not involving human participants)
How to access data: As stated in the manuscript, all codes of the study are published and openly available at the Zenodo registry. The study does not have other data.
When available: With publication

Supporting Documents
Document types: Statistical/analytic code
How to access documents: In the interest of research transparency and reproducibility, the analysis code used in this study has been made publicly available on GitHub and permanently archived on Zenodo under DOI 10.5281/zenodo.17688979.
When available: With publication

Additional Information
Who can access the data: Anyone requesting the data
Types of analyses: For cost-effective analysis on treatment of subclinical AF.
Mechanisms of data availability: With investigator support.
Any additional restrictions: R codes.
